# Comparative single-cell genomics of two uncultivated *Naegleria* species harboring *Legionella* cobionts

**DOI:** 10.1128/msphere.00352-25

**Published:** 2025-08-27

**Authors:** Jamie McGowan, Estelle S. Kilias, James Lipscombe, Elisabet Alacid, Tom Barker, Leah Catchpole, Seanna McTaggart, Sally D. Warring, Karim Gharbi, Thomas A. Richards, Neil Hall, David Swarbreck

**Affiliations:** 1 Earlham Institute, Norwich Research Park 455075, Norwich, United Kingdom; 2 Department of Biology, University of Oxford 6396, Oxford, United Kingdom; 3 School of Biological Sciences, University of East Anglia 6106, Norwich, United Kingdom; University of California Davis, Davis, California, USA

**Keywords:** Heterolobosea, amoeboflagellates, protists, Legionellaceae, secretion systems, intracellular pathogens, effectors

## Abstract

Amoeboflagellates of the genus *Naegleria* are free-living protists ubiquitously found in soil and freshwater habitats worldwide. They include the “brain-eating amoeba” *Naegleria fowleri*, an opportunistic pathogen that causes primary amoebic meningoencephalitis, a rare but fatal infection of humans. Beyond their direct pathogenicity, protists can also act as environmental reservoirs for intracellular bacterial pathogens, such as *Legionella* spp., to persist and multiply in the environment. In this study, we carried out single-cell genome sequencing of two uncultivated *Naegleria* species isolated from the River Leam in England. From single cells, we generated two highly complete *Naegleria* genomes. Phylogenetic analyses placed these species as close relatives of *Naegleria fultoni* and *Naegleria pagei*. Exploring *Naegleria* evolutionary genomics, we identified gene families encoding antistasin-like domains, which have been characterized as factors that inhibit coagulation in blood-feeding leeches. Antistasin-like domains were identified in all sequenced *Naegleria* species and their close relative *Willaertia magna,* yet are otherwise largely restricted to animal genomes. Significantly, we recovered highly complete bacterial genomes from each *Naegleria* single-cell sample. Phylogenomic analysis revealed that both bacteria belong to the Legionellaceae family. Both bacterial genomes encode comprehensive sets of secretion systems and effector arsenals. We identified putative *Legionella* effectors that resemble TAL (Transcription activator-like) effectors from plant pathogenic *Xanthomonas* spp. in terms of protein sequence and predicted structure, representing a potentially novel class of *Legionella* effectors. Our study highlights the advantages of single-cell environmental genomics approaches, which enable direct association of intracellular pathogens with their hosts to better understand the evolution of host-pathogen interactions.

**IMPORTANCE:**

Beyond their direct pathogenic potential, amoebae and other protists found in the environment can indirectly threaten human health by serving as reservoirs for intracellular bacterial pathogens to persist, evolve, and multiply in the environment. Despite their importance, protist-bacterial interactions remain poorly understood. In this study, we employed single-cell genomics to sequence the genomes of two uncultivated *Naegleria* amoebae, both harboring novel *Legionella* bacteria. From individual cells, we recovered highly complete eukaryotic and bacterial cobiont genome assemblies. Our work demonstrates the power of single-cell sequencing approaches in directly linking intracellular pathogens to their hosts to better understand the evolution of protist-bacterial interactions and the role that protists play in facilitating bacterial pathogens to persist long term in the environment.

## INTRODUCTION

*Naegleria* is a genus of free-living heterotrophic protists from the phylum Heterolobosea that are found in soil and freshwater habitats worldwide (1). *Naegleria* species are amoeboflagellates that can transform between amoeboid forms and rapidly moving flagellates or resting cysts. There are currently 47 recognized *Naegleria* species (2), but only *Naegleria fowleri—*the “brain-eating amoeba”—is known to infect humans. *N. fowleri* causes primary amoebic meningoencephalitis (PAM) in humans, a rare but fatal infection (>95% mortality rate) of the central nervous system. Infection occurs when contaminated water enters the nose (e.g., during swimming, bathing, or nasal irrigation), and *N. fowleri* invades the brain via the olfactory nerves and cribriform plate, where it causes severe tissue damage and hemorrhagic necrosis, generally resulting in death within 7–10 days post-infection (3). Like other *Naegleria* species, *N. fowleri* can persist at high temperatures (up to 45°C) (2), making warm bodies of water, hot springs, and poorly maintained water systems potential sources of exposure. With the ongoing effects of climate change and increasing global temperatures, there is growing concern that the abundance and geographic range of *N. fowleri* is expanding (4). To date, the genomes of four *Naegleria* species have been sequenced*—N. gruberi*, *N. fowleri*, *N. lovaniensis*, and *N. clarki* (5–8). *Naegleria* nuclear genomes are relatively small and gene-dense, with genome sizes ranging from 27 to 54 Mb, and have a low GC content in the 33%–37% range (5–9). Unlike most eukaryotes, which encode ribosomal RNA (rRNA) genes within their nuclear genomes, *Naegleria* rRNA genes are exclusively encoded on a circular extrachromosomal plasmid present at thousands of copies per cell (10). The availability of multiple *Naegleria* genome sequences has advanced our understanding of their evolutionary biology, metabolic diversity, and the pathogenic mechanisms of *N. fowleri*.

Amoeba and other protists pose a further threat to human health by serving as hosts for intracellular bacterial pathogens. By providing a niche for bacterial pathogens to persist and rapidly multiply in the environment, they increase the chance of transmission to humans (11). *Legionella* is a genus of intracellular gammaproteobacterial pathogens that cause Legionnaires’ disease in humans, a severe and potentially fatal form of pneumonia (12). *Legionella* exhibit a remarkably broad host range. In addition to humans, they infect a diverse array of distantly related protists in the environment, including members of the Amoebozoa, Ciliophora, and Heterolobosea phyla (13). The *Legionella* genus comprises at least 24 species that have been associated with human infection, with approximately 90% of Legionnaires’ disease attributed to a single species—*Legionella pneumophila* (12). *Legionella* virulence relies on a type IVb secretion system, termed the Dot/Icm (defective in organelle trafficking/intracellular multiplication) system, which translocates effector proteins into eukaryotic host cells to modulate host cellular processes and facilitate intracellular replication of the pathogen (14). *L. pneumophila* possesses a large arsenal of more than 300 effector proteins (approximately 10% of the entire proteome) that have been experimentally shown to be translocated into host cells via the Dot/Icm system (15, 16). Previous studies have demonstrated that many *Legionella* effectors contain eukaryotic domains—protein domains that are common in eukaryotic proteomes but typically absent in prokaryotes—such as ankyrin repeats, leucine-rich repeats, F-box, and U-box domains (16, 17). Intriguingly, evidence shows that many *Legionella* effector proteins were acquired via horizontal gene transfer (HGT) from their diverse protist hosts (17, 18). The acquisition and co-option of such host-derived genes enables the intracellular pathogen to subvert eukaryotic host cell functions and responses during infection. Thus, beyond serving as reservoirs for intracellular bacteria to multiply in the environment, the co-evolution of intracellular bacteria with protists in the environment may drive the evolution and emergence of novel human pathogens.

In this study, we report the single-cell genome sequencing of two uncultivated *Naegleria* species recovered during environmental single-cell sampling of the River Leam in England. From two single cells, we recovered two highly complete *Naegleria* nuclear genome assemblies, circular mitochondrial genomes, and extrachromosomal rDNA plasmids. Phylogenetic analysis of their 18S rRNA genes suggests that the two uncultivated *Naegleria* cells are, or are closely related to, *Naegleria fultoni* and *Naegleria pagei*. We carried out comparative genomic and phylogenomic analyses to explore the evolutionary genomics of *Naegleria* and related heterolobosean species. Significantly, from both *Naegleria* single-cell samples, we also recovered highly complete bacterial genomes. Phylogenomic analyses revealed that one of the bacterial species is an early diverging member of the Legionellaceae family, branching before named the *Legionella* species, while the other bacteria are most closely related to *Legionella lytica*, a known pathogen of *Naegleria* and other diverse amoeba. Both genomes encode Dot/Icm secretion systems and Type IVa pili, and one genome encodes a larger arsenal of virulence-related machinery, including Type I secretion system, Type II secretion system, Type IVa secretion system, Type Va secretion system, and flagellum-related machinery. While exploring putative effector proteins, we identified putative effectors in 14 *Legionella* genomes that resemble *Xanthomonas* TAL (Transcription activator-like) effectors in terms of protein sequence, repeat domains, and predicted structures. TAL effectors bind to host gene promoter sequences to directly modulate the expression of host genes to promote bacterial colonization (19). Thus, the presence of putative TAL effector-like proteins in *Legionella* genomes warrants further investigation to determine if they play a role in host-pathogen interactions. Our approach highlights the advantages of environmental single-cell sequencing in directly linking intracellular pathogens and cobionts to their hosts, unlike metagenomic or metabarcoding methods that rely on statistical co-occurrences. This enables deeper insights into the evolution and ecology of protist-bacterial interactions.

## RESULTS AND DISCUSSION

### Single-cell genome assemblies of two uncultivated *Naegleria* species

As part of an environmental single-cell genome sequencing experiment targeting heterotrophic protists, we recovered two cells that were later identified post-sequencing as belonging to the *Naegleria* genus. The two cells were recovered from surface water collected from the River Leam in Royal Leamington Spa (UK) in August 2022. The most recent assessment by the Environment Agency (2022) classified the river as having a “poor ecological status.” Collected water was prefiltered, concentrated, and incubated for 1 week in the presence of autoclaved barley grains prior to fluorescence-activated cell sorting (FACS). We refer to the two cells as *Naegleria* sp. PL0398 and *Naegleria* sp. PL0403. DNA from the single cells was amplified using multiple displacement amplification (MDA), and short-read sequencing was performed. Approximately 19.5 million and 47.7 million Illumina paired-end 150 bp reads were generated for *Naegleria* sp. PL0398 and *Naegleria* sp. PL0403, respectively.

*De novo* genome assembly followed by manual curation to separate *Naegleria* sequences from non-*Naegleria* sequences yielded a 41.4 Mb nuclear genome assembly for *Naegleria* sp. PL0398 with 5,607 scaffolds and an N50 of 16.8 kb (Table 1). *De novo* repeat annotation of *Naegleria* sp. PL0398 classified 3.3 Mb (7.9%) of the genome as repetitive, and genome annotation predicted 20,630 protein-coding genes with a BUSCO completeness of 84% (Table 1). The nuclear genome assembly of *Naegleria* sp. PL0403 was 36.7 Mb with 7,902 scaffolds and an N50 of 7.8 kb (Table 1). *De novo* repeat annotation of *Naegleria* sp. PL0403 classified 2.6 Mb (7.2%) of the genome as repetitive and genome annotation predicted 18,171 protein-coding genes with a BUSCO completeness of 78.4% (Table 1). The GC content of both genomes was low at 33.9% and 34.8% for *Naegleria* sp. PL0398 and *Naegleria* sp. PL0403, respectively (Table 1). Analysis post-curation using BlobTools confirmed both *Naegleria* assemblies were free from contamination (Fig. S1).

**TABLE 1:** *Naegleria* single-cell genome assembly statistics

Parameter	*Naegleria* sp. PL0398	*Naegleria* sp. PL0403
Nuclear genome		
Total length	41,391,420 bp	36,653,337 bp
Scaffolds	5,607	7,902
N50	16,805 bp	7,812 bp
GC content	32.88%	34.78%
Repetitive elements	3,272,173 bp (7.91%)	2,647,036 bp (7.22%)
Protein-coding genes	20,630	18,171
Single-exon genes	11,932 (57.84%)	10,906 (60.02%)
Average CDS length	1,457 bp	1,390 bp
BUSCO completeness (protein mode; eukaryota_odb10)	84% complete 81.6% complete single copy 2.5% complete duplicated 5.9% fragmented 10.1% missing	78.4% complete 75.7% complete single copy 2.7% complete duplicated 8.2% fragmented 13.4% missing
Mitochondrial genome		
Total length	49,712 bp	49,761 bp
Protein-coding genes	42	42
Open reading frames	4	4
tRNA genes	21	22
Extrachromosomal rRNA plasmid		
Total length	13,425 bp	15,294 bp

For both species, we also recovered circular mitochondrial genome assemblies. The mitochondrial genome of *Naegleria* sp. PL0398 was 49,712 bp in length with 42 protein-coding genes, four open reading frames (ORFs) with unknown function, 21 tRNA genes, and the large and small subunit rRNA genes (Fig. S2A; Table 1). The mitochondrial genome of *Naegleria* sp. PL0403 was 49,761 bp in length with 42 protein-coding genes, 4 ORFs with unknown function, 22 tRNA genes, and the large and small subunit rRNA genes (Fig. S2A; Table 1). The gene content and gene order of the mitochondrial genomes are conserved with other *Naegleria* species (20, 21).

We also recovered extrachromosomal rDNA plasmids from both *Naegleria* assemblies, which encode the rRNA genes. The plasmids were 13,425 bp and 15,294 bp in length for *Naegleria* sp. PL0398 and *Naegleria* sp. PL0403, respectively (Fig. S2B; Table 1), and both encode single copies of the 18S, 5.8S, and 28S rRNA genes. The *Naegleria* sp. PL0398 plasmid encodes a single ORF (orf566) (Fig. S2B), which was annotated with two copies of the Pfam domain “Zinc-binding loop region of homing endonuclease” (PF05551). The *Naegleria* sp. PL0403 plasmid encodes six ORFs (Fig. S2B), including two identical copies of orf110. Four of the ORFs (orf115, orf122, and the two copies of orf110) contain no annotated functional domains. orf203 and orf223 were both annotated with the Pfam domain “Zinc-binding loop region of homing endonuclease” (PF05551). The 18S rRNA sequence of *Naegleria* sp. PL0398 is 99.8% identical to that of *Naegleria pagei* (DQ768714.1), while the 18S rRNA of *Naegleria* sp. PL0403 is 100% identical to that of *Naegleria fultoni* (DQ768719.1). Maximum-likelihood phylogenetic analysis of the 18S rRNA sequences placed the *Naegleria* sp. PL0398 sequence within a monophyletic clade containing three *N. pagei* sequences (Fig. S3). The *Naegleria* sp. PL0403 sequence was placed within a monophyletic clade containing two *N. fultoni* sequences (Fig. S3). Thus, these data suggest that the two uncultivated environmental species are, or are closely related to, *N. pagei* and *N. fultoni. N. pagei* has previously been reported in Europe, North America, and Asia and has a reported maximum temperature range of 37°C (2). Whereas *N. fultoni* has been reported in Asia and Australia and has a reported maximum temperature range of 35°C (2). Thus, our work extends the geographic range of *N. fultoni* to Britain.

### Comparative genomics of *Naegleria* and other Heterolobosea

**Fig 1:**
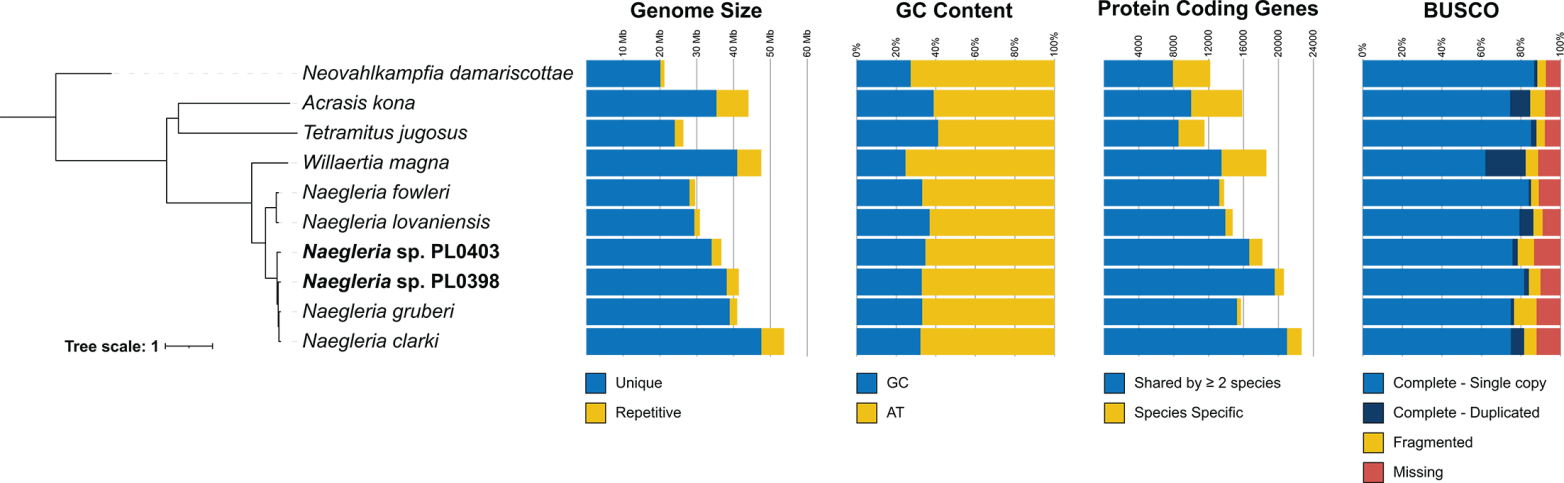
Phylogenomic analysis of Heterolobosea. Bayesian phylogenetic analysis was conducted on a concatenated alignment of 152 BUSCO proteins (76,279 amino acid sites after trimming) using PhyloBayes-MPI under the CAT-GTR model. The phylogeny was rooted using *Neovahlkampfia damariscottae* as an outgroup. All branches have full statistical support (i.e., posterior probability of 1). Bar plots show the genome sizes, % GC content, number of protein-coding genes, and estimated BUSCO completeness for each genome.

To characterize genomic evolution across Heterolobosea, we conducted comparative genomic analyses of previously sequenced *Naegleria* species, including *N. clarki*, *N. fowleri*, *N. gruberi*, and *N. lovaniensis*, along with related species from the Heterolobosea phylum, including *Willaertia magna*, *Acrasis kona*, *Tetramitus jugosus*, and *Neovahlkampfia damariscottae*. We generated genome annotations for *T. jugosus* and *N. damariscottae*, which were previously deposited without annotations (22, 23), using the same approaches that we applied to annotate *Naegleria* sp. PL0398 and *Naegleria* sp. PL0403 (see Materials and Methods). The assembly sizes, repeat content, number of predicted proteins, and BUSCO completeness of the two uncultivated *Naegleria* species were in line with published *Naegleria* genomes that were sequenced from bulk cultures (Fig. 1), suggesting that our single-cell genome assemblies are highly complete. We identified a moderate positive correlation between Heterolobosean genome size and repeat content (*R*^2^ = 0.59) and a strong positive correlation between genome size and the number of protein-coding genes (R^2^ = 0.76). We note that 20 out of the 255 BUSCO proteins in the eukaryota_odb10 data set are missing from all six *Naegleria* species in our data set (Table S1). This suggests that these BUSCO genes have been lost from this lineage rather than being absent due to issues with genome incompleteness.

Phylogenomic analysis was performed on a concatenated alignment of 152 BUSCO proteins (76,279 amino acids) that were complete and single copy in at least nine out of ten species. Bayesian phylogenetic reconstruction using PhyloBayes-MPI with the CAT-GTR model yielded a robust species phylogeny with all branches receiving full support (i.e., posterior probability of 1) (Fig. 1). The *Naegleria* genus was divided into two groups—a lineage containing *N. fowleri* and the closely related *N. lovaniensis*, and a second lineage containing the other four *Naegleria* species, with *N. clarki* and *N. gruberi* grouped as sister species (Fig. 1).

**Fig 2:**
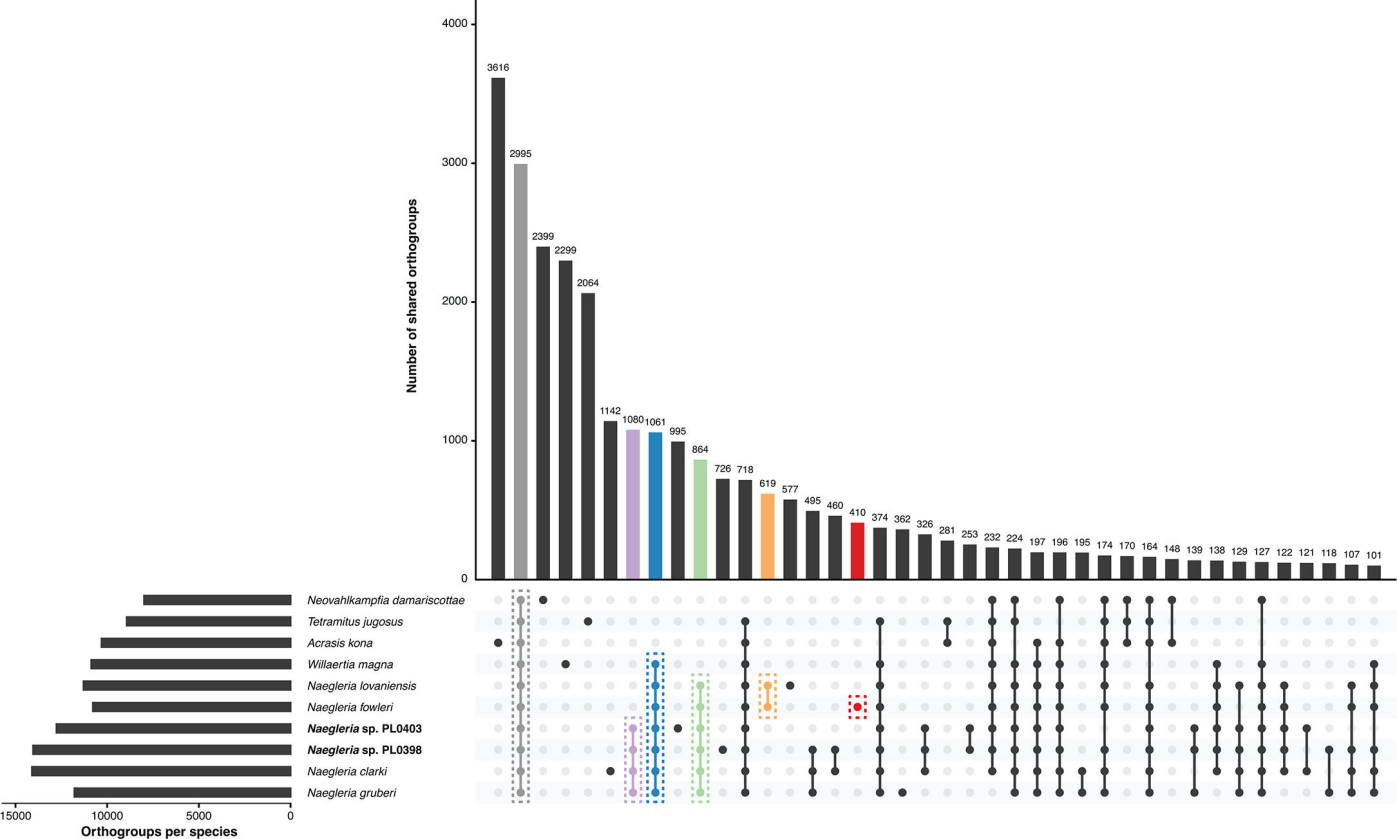
UpSet plot showing the conservation of orthogroups across Heterolobosea. The total number of orthogroups encoded by each genome is indicated by the horizontal bar plot. The number of orthogroups shared between genomes is indicated by the vertical bar plot. Orthogroup comparisons discussed in the text are colored. The 40 largest intersections are displayed.

To explore the evolution of heterolobosean gene families, we identified orthogroups using OrthoFinder. 2,995 orthogroups were conserved across all 10 heterolobosean genomes (Fig. 2), of which 1,063 were present as single copies in all 10 genomes. *A. kona* encodes the largest number of unique orthogroups with 3,616 species-specific orthogroups (Fig. 2), representing approximately 36.9% of its entire proteome, in line with previous reports (24). The number of orthogroups unique to other non-*Naegleria* heterolobosean species was also high (Fig. 2), with 2,399 orthogroups unique to *N. damariscottae* (35% of proteome), 2,299 to *W. magna* (27.7% of proteome), and 2,064 to *T. jugosus* (25.9% of proteome), highlighting the divergence between these species and their closest sequenced relatives (Fig. 1). 1,061 orthogroups were uniquely conserved between the *Naegleria* genus and its closest relative *W. magna*, while 864 orthogroups were uniquely conserved within *Naegleria* (Fig. 2). Within *Naegleria*, 1,080 orthogroups were uniquely conserved within the lineage containing *N. clarki*, *N. gruberi*, *Naegleria* sp. PL0398, and *Naegleria* sp. PL0403, whereas 619 orthogroups were unique to *N. fowleri* and its closest relative *N. lovaniensis* (Fig. 2). The number of orthogroups encoded per heterolobosean genome ranged from 7,985 (*N. damariscottae*) to 14,101 (*N. clarki*) (Fig. 2). *N. fowleri* encodes the smallest number of orthogroups among *Naegleria,* at 10,782 orthogroups. In all, 410 orthogroups were identified as being unique to *N. fowleri,* which includes 549 member genes. The number of *N. fowleri*-specific genes is comparable to previous reports (25). Orthogroups unique to *N. fowleri* were largely made up of singletons or small gene families (mean orthogroup size of 1.3 genes). Only 115 of these genes (21%) were annotated with a Pfam domain (Table S2), and only 17 were predicted to be secreted. Future characterization of genes unique to *N. fowleri* that lack functional annotations will provide better insights into *N. fowleri* virulence and cell biology.

**Fig 3:**
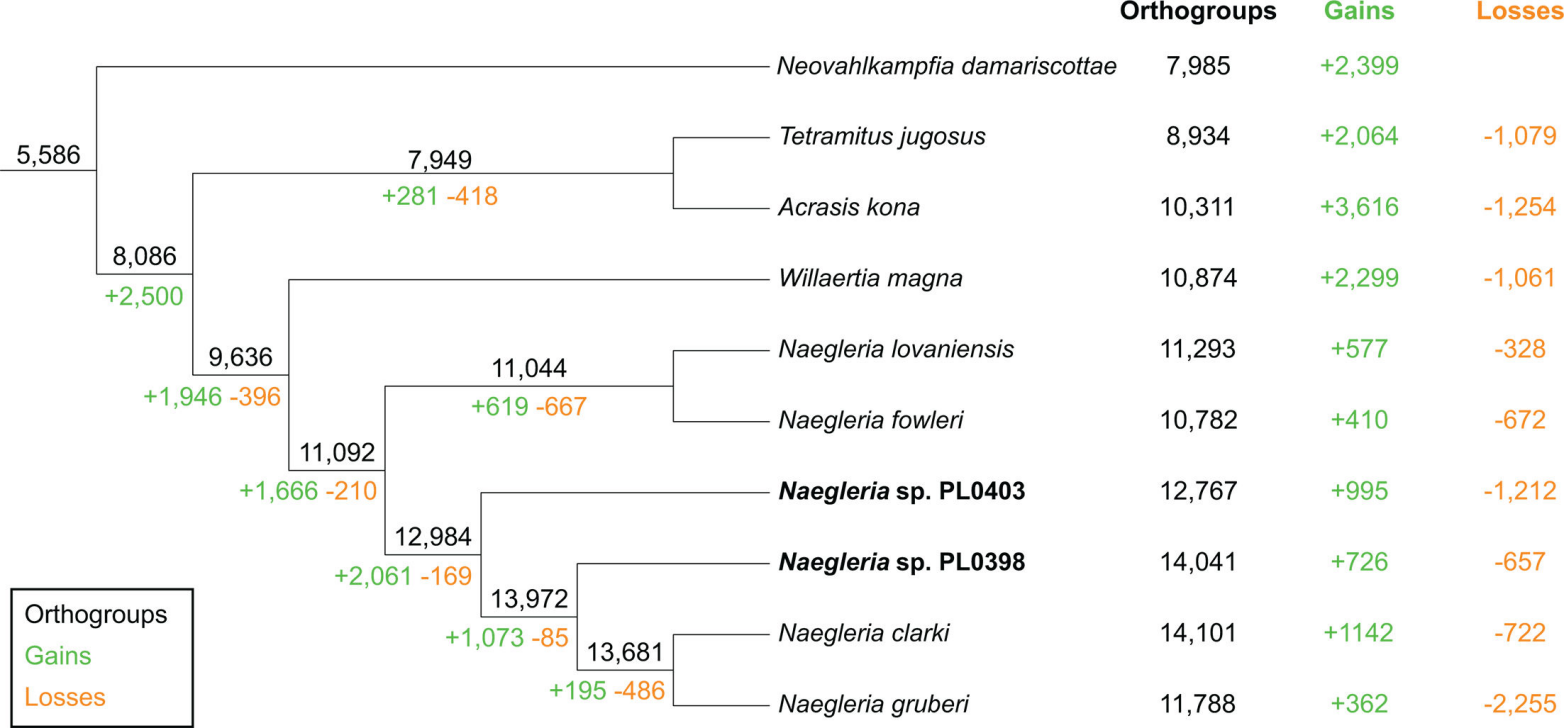
Patterns of orthogroup gains and losses across Heterolobosea. Orthogroups were mapped onto the species phylogeny (from Fig. 1), and gains and losses were inferred under Dollo parsimony using Count. Numbers at the tips represent the total number of orthogroups, as well as the gains and losses inferred for extant species. Numbers along the branches indicate inferred ancestral state reconstructions.

We mapped orthogroups onto the species phylogeny and performed ancestral state reconstruction under Dollo parsimony to infer orthogroup gains and losses. This analysis inferred that the most recent common ancestor of sampled heterolobosean species encoded 5,586 orthogroups (Fig. 3). The most recent common ancestor of *Naegleria* was inferred to encode 11,092 orthogroups (Fig. 3). The largest number of orthogroup losses was inferred in *N. gruberi*, which lost 2,255 orthogroups relative to its most recent common ancestor with *N. clarki*, which was 6.3-fold greater than the number of gains of 362 orthogroups (Fig. 3). We inferred that *N. fowleri* lost 672 orthogroups since its most recent common ancestor with *N. lovaniensis* (Fig. 3). In *N. lovaniensis,* these 672 orthogroups include 905 member genes that were annotated with 303 diverse Pfam functional domains (Table S2). Most of these Pfam domains are found in other *N. fowleri* orthogroups; however, we identified 78 Pfam domains from this set of lost orthogroups that are not encoded by *N. fowleri* (Table S2).

### *Naegleria* and *Willaertia* encode proteins containing antistasin-like domains

Intriguingly, when examining orthogroups unique to the *Naegleria* genus and its closest relative *W. magna*, we identified proteins annotated with antistasin-like domains (IPR004094). Antistasin-like proteins are a family of serine protease inhibitors best known as factors that inhibit hemostasis (blood clotting) during blood feeding by leeches (26). These proteins are composed of one or more repeats of an antistasin-like domain, a cysteine-rich repeat stabilized by multiple disulfide bonds. Antistasin-like proteins have also been reported in non-bloodfeeding animals where their function is unknown (26).

We identified 59 proteins in our data set that were annotated with antistasin-like domains (Table S3). Each *Naegleria* species encodes between five and nine proteins containing the domain, and *W. magna* encodes 17 proteins. OrthoFinder grouped these 59 protein sequences into 10 different orthogroups (Table S3). The number of antistasin-like domains per protein ranges from a single copy up to 11 copies (mean 2.6 copies per protein). While these proteins encoded the cysteine-rich antistasin-like domains (IPR004094), they typically contained fewer cysteine residues overall when compared to characterized antistasin-like proteins (26). N-terminal signal peptides were predicted in 51 (86.4%) proteins, suggesting that they are secreted (Table S3). The taxonomic distribution of antistasin-like domains is unusual. Of the 3,582 proteins annotated with the domain (IPR004094) in the InterPro database, 3,504 (97.8%) belong to eukaryotes, of which 3,473 (99.1%) are from Opisthokonta. Within Opisthokonta, these proteins are restricted to Metazoa (99.6%) and the related protist lineages Choanoflagellata and Ichthyosporea. The presence of antistasin-like proteins in *Naegleria* and *Willaertia* genomes is therefore unexpected. Comparison of *Naegleria* and *Willaertia* sequences with other antistasin-like proteins, including the archetypal antistasin from *Haementeria officinalis* (Mexican leech), revealed that sequence similarity is largely restricted to the cysteine-rich repeat domains, which prevents detailed phylogenetic analysis. Thus, the evolutionary history of *Naegleria* and *Willaertia* antistasin-like domain-containing proteins is unclear. Their presence in non-pathogenic *Naegleria* and *Willaertia* species suggests they may be involved in other biological processes unrelated to the inhibition of blood clotting. Further investigation is needed to identify the target of these putative protease inhibitors and determine their functional roles.

### Novel Legionellaceae bacteria identified as putative cobionts of *Naegleria*

From both initial *Naegleria* single-cell genome assemblies, we identified the presence of *Legionella*-like genomes. We manually curated each bacterial genome to separate it from *Naegleria* sequences. The bacterial genome associated with *Naegleria* sp. PL0398 (*Legionella* sp. PL0398) was 1.62 Mb in length with a GC content of 33.04% and 1,432 predicted proteins (Table 2). CheckM2 estimated that the assembly was 80% complete with virtually no detected contamination (0.14%) (Table 2). The genome of *Legionella* sp. PL0398 encodes 29 tRNA genes (for 17 of the standard amino acids) and the 23S, 16S, and 5S rRNA genes. This assembly is considered a medium-quality draft SAG according to the MISAG standards (≥50% completeness and <10% contamination) (27). The bacterial genome associated with *Naegleria* sp. PL0403 (*Legionella* sp. PL0403) was 3.1 Mb in length with a GC content of 39.73% and 2,893 predicted proteins (Table 2). CheckM2 estimated that the assembly was 94.77% complete with virtually no detected contamination (0.19%) (Table 2). The genome of *Legionella* sp. PL0403 encodes 35 tRNA genes (for 18 of the standard amino acids), a tmRNA, and the 23S, 16S, and 5S rRNA genes. This assembly is considered a high-quality draft SAG according to the MISAG standards (>90% completeness, <5% contamination, presence of the 23S, 16S, and 5S rRNA genes, and tRNA genes for ≥18 out of 20 amino acids) (27).

**TABLE 2:** *Legionella* genome statistics^*a*^

Parameter	*Legionella* sp. PL0398	*Legionella* sp. PL0403	Legionellaceae range	Legionellaceae mean
Genome size	1,624,929 bp	3,105,639 bp	1,127,886–5,241,559 bp	2,869,772 bp
GC content	33.04%	39.73%	33.51–50.88%	40.33%
Protein-coding genes	1,432	2,893	1,041–4,595	2,571
Average CDS length	933 bp	914 bp	588–1,082 bp	968 bp
CheckM2 completeness	80.01%	94.77%	51.86%–100%	95.22%
CheckM2 contamination	0.14%	0.19%	0%–16.77%	0.95%
tRNA genes (amino acids)	29 (17)	35 (18)	—	—
rRNA genes	23S, 16S, 5S	23S, 16S, 5S	—	—
MISAG quality	Medium	High	—	—
Secretion and related systems	Dot/Icm, T4aP	Dot/Icm, T1SS, T2SS, T4ASS, T5aSS, T4aP, flagellum	—	—

^*a*^ “Range” and “mean” are calculated from 165 bacterial genomes in our data set, which excludes the two novel *Naegleria* associated assemblies reported here, as well as the outgroup *Coxiella burnetti* and the symbiotic *Legionella polyplacis* which has a highly reduced genome (Fig. 4). —, not applicable.

**Fig 4:**
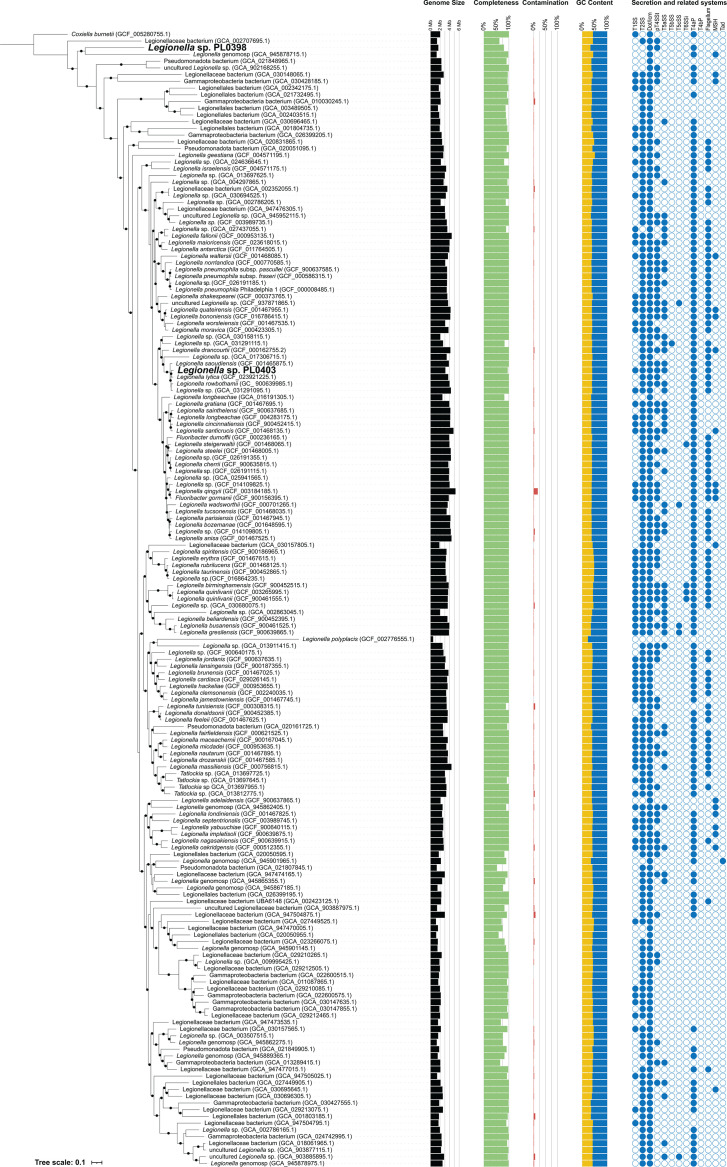
Phylogenomic analysis of Legionellaceae genomes. Partitioned maximum likelihood phylogenomic reconstruction was performed using IQ-TREE on a concatenated alignment of 184 BUSCO proteins (47,837 amino acid sites after trimming) from 168 Legionellaceae genomes, along with *Coxiella burnetii,* which was included as an outgroup. The two novel bacterial genomes recovered from *Naegleria* single-cell sequencing (*Legionella* sp. PL0398 and *Legionella* sp. PL0403) are highlighted. Solid black dots indicate branches with ≥90% ultrafast bootstrap support and ≥90% SH-aLRT support. Genome assembly size, estimated completeness (CheckM2), estimated contamination (CheckM2), % GC content, and detected secretory- and virulence-related machinery are indicated.

To compare the novel *Legionella*-like genomes with reference *Legionella* genomes, we retrieved 166 species representative genomes for the Legionellaceae family from the GTDB database, along with *Coxiella burnetii,* which served as an outgroup species. For consistency, we reannotated all bacterial genomes in our data set using Prokka. The genome assembly statistics of our two *Legionella* genomes were within the ranges observed in other members of the Legionellaceae family (Table 2; Fig. 4). A partitioned maximum-likelihood phylogenomic analysis of 184 BUSCO proteins yielded a robust and highly supported phylogeny (Fig. 4). *Legionella geestiana* was placed as the most basal named *Legionella* species (Fig. 4) as previously reported (28). *Legionella* sp. PL0398 was placed as an early-diverging taxon, branching before named the *Legionella* species (Fig. 4). *Legionella* sp. PL0403 was grouped as a sister to *Legionella lytica* (Fig. 4). The 16S rRNA genes of *Legionella* sp. PL0403 and *L. lytica* are 98.8% identical, but their genomes only share 88.6% average nucleotide identity (ANI) based on a 2,045,567 bp alignment (corresponding to 65.9% and 55.2% of their respective genomes). *L. lytica*, previously named *Sarcobium lyticum*, is an obligate intracellular bacterial parasite of diverse protists, including *N. gruberi*, *Acanthamoeba*, and *Hartmannella*, that was originally isolated from soil (29). Other members of this clade include *Legionella rowbothamii* and *Legionella saoudiensis* (Fig. 4), known pathogens of amoeba isolated from a factory liquefier tower (30) and sewage water (31), respectively. Notably, 58% of Legionellaceae genomes included in our data set are from yet-to-be-described species (Fig. 4), which were obtained via metagenomics and thus cannot be linked to their hosts. The extensive prior sampling of *Legionella* and amoebae (13) supports that the two novel Legionellaceae recovered in this study are bona fide *Naegleria* cobionts, rather than the result of passive sampling. This highlights the advantages of our single-cell environmental genomics approach, which enables the direct association of intracellular pathogens with their hosts.

### *Legionella* secretion and virulence-related systems

**Fig 5:**
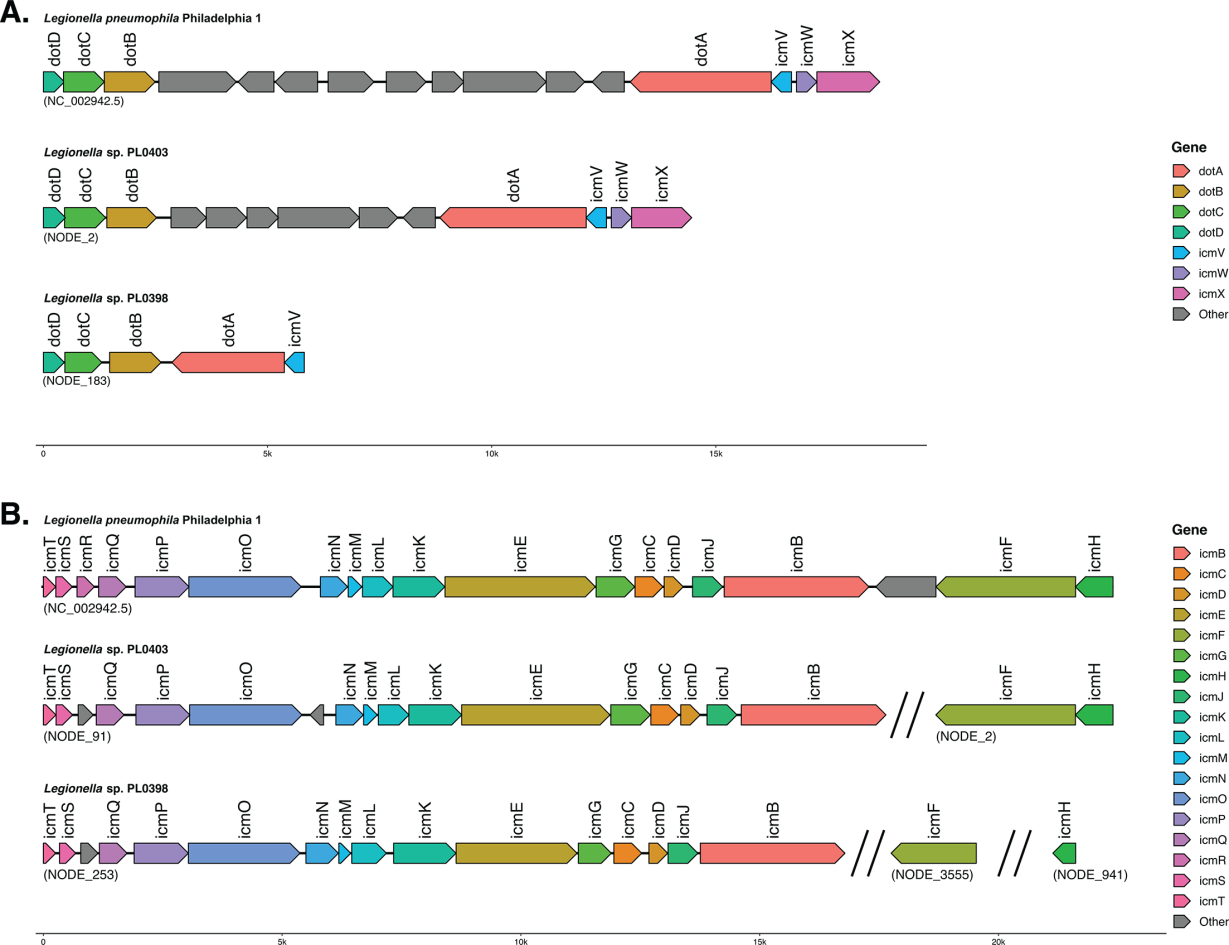
Organization of the Dot/Icm secretion system region I (**A**) and region II (**B**). The Dot/Icm secretion system of *Legionella pneumophila* is also shown as a reference. Note that the Dot/Icm region II is fragmented across multiple contigs in *Legionella* sp. PL0398 and *Legionella* sp. PL0403.

Given their importance in virulence and intracellular lifestyles, we searched for secretion systems in both novel *Legionella* genomes. Through manual annotation, we identified Dot/Icm secretion systems in both genomes. As observed in all *Legionella* species, components of the Dot/Icm secretion system are encoded in two separate genomic regions (Fig. 5). Dot/Icm secretion system region I encodes genes for dotA, dotB, dotC, dotD, icmV, icmW, and icmX (Fig. 5A). We identified all Dot/Icm region I components in *Legionella* sp. PL0403, which shared collinearity with *L. pneumophila,* with fewer gene insertions between dotA and dotB (Fig. 5A). dotA and dotB were immediately adjacent to each other in *Legionella* sp. PL0398 (Fig. 5A). We did not identify genes for icmW and icmX in any region of the genome assembly; however, it is possible that this is an artifact of genome incompleteness. Dot/Icm secretion system region II encodes genes for icmB-H and icmJ-T (Fig. 5B). All components of region II were identified in *Legionella* sp. PL0403, except for icmR, which was previously reported to be unique to *L. pneumophila* and the closely related *L. norrlandica* (14). icmF and icmH were located on a separate contig due to assembly fragmentation (Fig. 5B). All components of region II were also identified in *Legionella* sp. PL0398, except for icmR. Similarly, icmF and icmH were located on two separate contigs due to assembly fragmentation (Fig. 5B). We also manually annotated components of the Dot/Icm in the other 167 bacterial genomes in our data set. Most genomes (85%) contained all seven expected components of region I, and most (80%) contained all 17 expected components of region II (Table S4). Genomes with missing components of the Dot/Icm system typically had low completeness and/or were excessively fragmented (Table S4). Genomes with missing region I components typically had a complete or near-complete set of region II components, or vice versa. Thus, we considered the Dot/Icm to be present in 168 out of 169 bacterial genomes in our data set. The only genome lacking the Dot/Icm was *Legionella polyplacis* (Table S4).

Furthermore, we performed automated annotation of secretion systems in all 169 bacterial genomes in our data set using MacSyFinder to find additional secretion and related systems. We ran MacSyFinder in “ordered_replicon” mode, which requires component genes to be co-localized for a system to be considered present (32). In both novel *Legionella* genomes associated with *Naegleria*, we identified genes encoding Type IVa pili (T4aP). Type IV pili have been implicated in a broad range of cellular functions linked to pathogenesis, including host-cell adhesion, biofilm formation, motility, and DNA transformation (33). T4aP machinery is highly conserved across Legionellaceae and was identified in 135 out of 169 (79.9%) genomes in our data set (Fig. 4; Table S4). In addition, we identified Type I secretion system (T1SS), Type II secretion system (T2SS), Type IVa secretion system (T4aSS), and Type Va secretion system (T5aSS) genes in *Legionella* sp. PL0403, making it one of the most equipped *Legionella* species in terms of secretion machinery (Fig. 4; Table S4). T2SS was highly conserved across Legionellaceae and identified in 155 genomes (91.7%) (Fig. 4; Table S4). T4aSS was identified in 74 genomes (43.8%), T1SS in 72 genomes (42.6%), while T5aSS was identified in only 53 genomes (31.4%) (Fig. 4; Table S4). Furthermore, we identified flagellum-related genes in *Legionella* sp. PL0403, which were found in 77 genomes (45.6%) in our data set (Fig. 4; Table S4). Flagella play an important role in *Legionella* virulence by providing motility to enhance host cell contact, facilitating efficient invasion, and promoting dispersal to new hosts following host cell lysis (34). In *Legionella* sp. PL403, we also identified the presence of a HipBST toxin-antitoxin (TA) system (Table S4). HipBST is a tripartite TA system involving the toxin HipT (kinase), the antitoxin HipS, and HipB, which is involved in regulation of the system (35). The HipBST TA system is highly conserved across Legionellaceae and was identified in 85 genomes (50.9%) in our data set. It is not present in *Legionella* sp. PL0398. Interestingly, in 34 genomes (20.1%), both HipS and HipT are encoded by a single ORF (Table S4), similar to the HipA from the HipBA system in *Escherichia coli*. In these cases, the fused HipST ORF, and also HipB, has undergone significant sequence divergence (Table S4). The only species without any identified secretion or related machinery was *Legionella polyplacis*, a louse symbiont with a highly reduced genome (Fig. 4; Table S4), as previously reported (36). The genomic features encoded by *Legionella* sp. PL0398 and *Legionella* sp. PL0403 are consistent with intracellular lifestyles.

### Putative *Legionella* effectors resembling TAL effectors

To annotate putative effectors, we searched each of the 169 proteomes for previously reported eukaryotic domains that have been associated with *Legionella* effectors (17). On average, each Legionellaceae genome encodes 23 of the previously reported eukaryotic domains (Table S5). The genome of *Legionella* sp. PL0398 encodes 32 proteins containing 11 different eukaryotic domains (Table S5). The genome of *Legionella* sp. PL0403 encodes 35 proteins containing 23 eukaryotic domains (Table S5). Eukaryotic domains found in both genomes include those that are commonly found in *Legionella* genomes, such as ankyrin repeats, leucine-rich repeats, and U-box domains (Table S5).

Upon investigating InterPro domains that were poorly conserved across Legionellaceae, we identified protein sequences annotated with “TAL effector repeats” (IPR005042). Transcription activator-like (TAL) effectors, best studied in plant pathogenic bacteria such as *Xanthomonas*, bind to host gene promoters to directly modulate the expression of specific host genes to promote bacterial colonization (19). These effectors have a modular structure, including secretion signals, nuclear localization signals, and tandemly arranged copies of TAL effector repeats which mediate base-specific interactions with DNA. To our knowledge, TAL-like effectors have not been reported in *Legionella*. We identified 19 protein sequences from 14 genomes in our data set that were annotated with TAL effector repeats (Table S6). They were found in the genomes of nine unnamed species (including *Legionella* sp. PL0398) and five named species—*L. jamestowniensis*, *L. maceachernii*, *L. quateirensis*, *L. worsleiensis*, and *L. yabuuchiae*. All 19 proteins were predicted to localize to the nucleus by DeepLoc, and nuclear localization signals were identified in 14 proteins (Table S6). The number of TAL effector repeats identified by InterPro was low, ranging from a single copy to 11 copies per protein sequence (mean 4.8) (Table S6). For comparison, *Xanthomonas* TAL effectors typically contain 17 repeat copies (19). We note, however, that the detected *Legionella* repeats were generally divergent compared to those found in *Xanthomonas* and from the InterPro domain (IPR005042). Through manual analysis, we were able to identify additional repeat copies, increasing the number of repeats from 3 to 13 copies per protein (mean 7.3) (Table S6). As further evidence that the putative *Legionella* TAL-like effectors may be bona fide effectors, 16 of the 19 proteins were predicted to be Type IV secreted effectors by T4Sepp (Table S6), a deep learning classifier based on pretrained language models.

**Fig 6:**
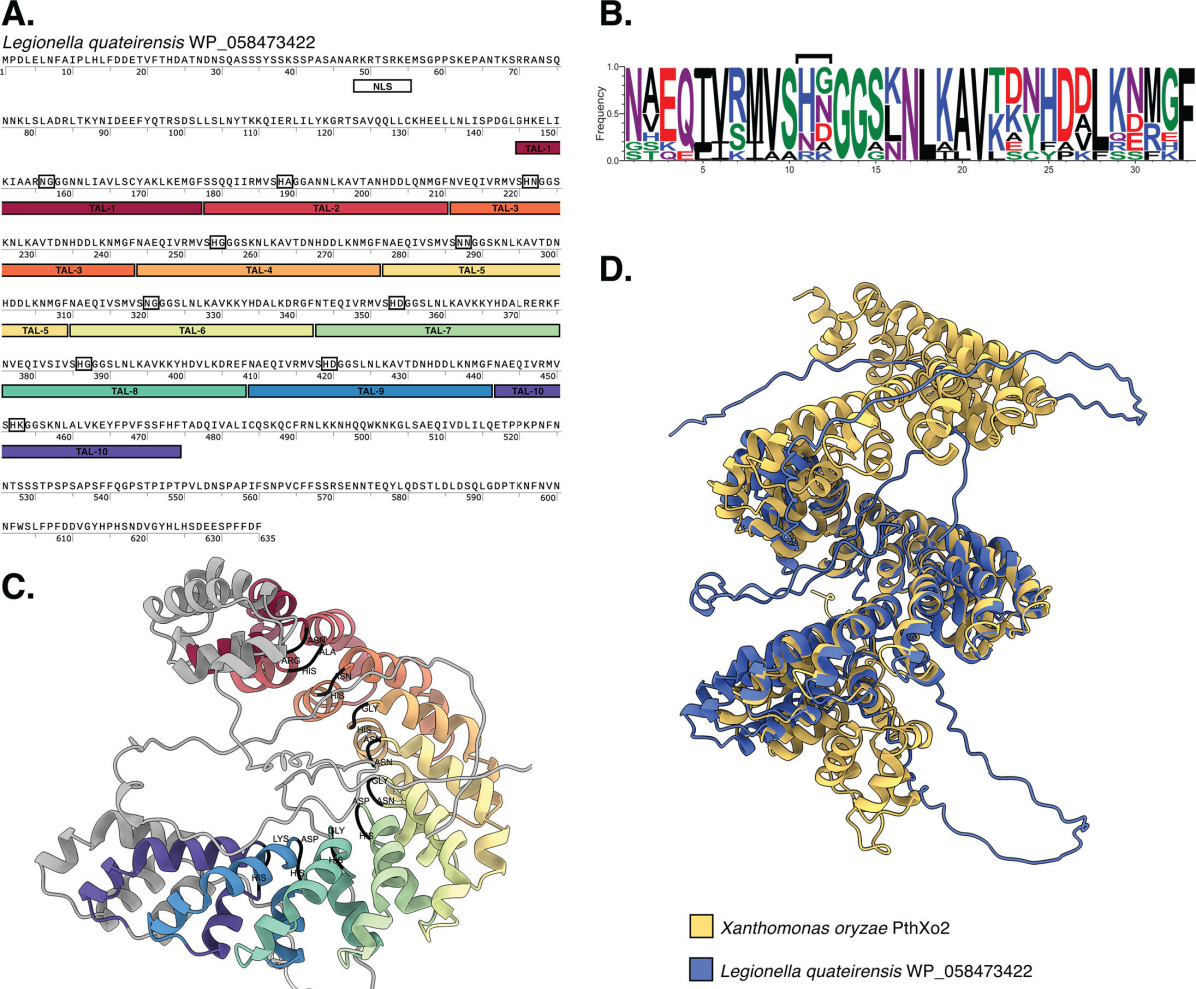
Putative *Legionella quateirensis* effector resembling TAL-like effectors. (**A**) Protein sequence of putative effector of *Legionella quateirensis* (protein ID WP_058473422). Repeat copies of TAL-like repeats are highlighted (TAL-N) along with a putative nuclear localization signal (NLS). Residues predicted to specify DNA binding are indicated (corresponding to positions 11 and 12 of each TAL-like repeat). (**B**) Sequence logo showing the consensus sequence of the 10 TAL-like repeats. The height of each amino acid corresponds to its frequency within the 10 TAL-like repeat copies. Residues predicted to specify DNA binding (positions 11 and 12) are indicated. (**C**) Predicted structure of putative *Legionella quateirensis* effector zoomed in on the TAL-like repeats. Each TAL-like repeat is colored as per Fig. 6A. Residues predicted to specify DNA binding are colored black and labeled. (**D**) Predicted structure of putative *Legionella quateirensis* effector aligned against a characterized TAL effector PthXo2 of the rice pathogen *Xanthomonas oryzae*. The two protein sequences only share 25% sequence identity, but their predicted structures have a template match score (TM-score) of 0.74.

We focused on a putative *Legionella* TAL-like effector from a named species to characterize further—a protein sequence from *L. quateirensis* (corresponding to NCBI RefSeq protein sequence WP_058473422). Manual analysis of this sequence identified 10 complete copies (Fig. 6A) of a highly conserved repeat 33 amino acids in length (Fig. 6B). The sequence composition of the repeat domains resembles TAL repeats from experimentally characterized TAL-effectors (19, 37), including two conserved glycine residues (positions 13 and 14 in Fig. 6B). The two preceding residues (positions 11 and 12 in Fig. 6B) are variable and resemble repeat-variable diresidues (RVDs) from characterized TAL-effectors, which specify DNA binding (37). The sequence also encodes a putative nuclear localization signal (Fig. 6A). We predicted the structure of the protein using AlphaFold 3 (Fig. 6C; Fig. S4). Like characterized TAL effectors, each of the TAL-like repeats was predicted to form two-helix bundles connected by a short loop that contains the RVD-like residues (Fig. 6C). The TAL-like repeats were predicted to associate to form a superhelix structure (Fig. 6C). The core of the protein structure, including the TAL-like repeats, was predicted with high/very high confidence (Fig. S4). In characterized TAL effectors, the superhelix structure wraps around its DNA target with the RVD making base-specific contact (37). In our predicted structure, the short loops containing the variable residues at positions 11 and 12 of each TAL-like repeat are positioned internally within the superhelix structure (Fig. 6C), resembling RVDs from known TAL effectors. Using Foldseek, we compared the predicted structure to that of PthXo2, a characterized TAL effector of the rice pathogen *X. oryzae*. This revealed that, while their protein sequences were only 25% identical, their structures have a TM-score (template match score) of 0.74 (Fig. 6D), which indicates significant structural similarity and may imply a shared functional mechanism. TAL effector-like proteins were also previously reported in *Mycetohabitans* species (bacterial endosymbionts of fungi) (38) and an uncultured bacterial lineage related to *Coxiella* that is associated with the marine choanoflagellate *Bicosta minor* (39). Their presence in *Legionella* genomes warrants further investigation to assess their potential role in pathogen-host interactions.

### Conclusion

Here, we carried out single-cell genome sequencing of two uncultivated *Naegleria* species recovered during environmental water sampling of the River Leam in England. From single cells, we generated genome assemblies that are comparable in completeness and gene content to existing *Naegleria* genome assemblies derived from bulk cultures. Comparative genomics across the Heterolobosea phylum revealed extensive lineage-specific gene content and identified protein families containing antistasin-like domains in *Naegleria* and *Willaertia* that are otherwise largely restricted to animal genomes. From the same single-cell samples, we also recovered two novel Legionellaceae genomes that encode comprehensive sets of secretion-related systems and effector arsenals. Novel putative effectors were identified that resemble TAL-like effectors in terms of protein sequence, repeat domains, and predicted structure, representing a potentially novel class of *Legionella* effectors. The combination of single-cell sampling, bacterial gene content, and prior extensive sampling of *Legionella* in diverse amoebae supports a cobiont lifestyle between each bacterium and *Naegleria*. Our findings demonstrate the potential of environmental single-cell sequencing to directly associate intracellular pathogens and cobionts with their hosts and to better understand the evolution of protist-bacterial interactions in natural ecosystems.

## MATERIALS AND METHODS

### Sampling and cell sorting

Surface water (~1 m) was collected from the River Leam (52.287295, −1.547563), Royal Leamington Spa (UK) in August 2022. Initially, the sample was pre-filtered to remove larger debris and then concentrated using different polycarbonate filter pore sizes (Millipore) to obtain different concentrated subsamples of different protist size ranges (10–40 µm and 0.8–5 µm). The subsamples were supplemented with 2–3 autoclaved barley grains to support heterotrophic/mixotrophic growth via bacteria increase and incubated for 1 week prior to cell sorting. Single-cell nuclei were stained with 1xSybrGreen for 10 minutes and sorted into 96-well microplates (pre-filled with 5 µL autoclaved/sterile filtered media), using fluorescence-activated cell sorting (FACS; flow rate = 1) and selecting against chlorophyll a-positive but SybrGreen-positive cells. After cell sorting, 10 µL of RLT-plus lysis buffer was added to the wells, and the plate was frozen at −80C until further processing.

### Whole-genome amplification, library preparation, and sequencing

A modified G&T-seq protocol (40) was carried out as follows. Using a magnetic separator, Dynabeads MyOne Streptavidin C1 (Invitrogen) beads were washed according to the manufacturer’s guidance and then incubated with 2× binding & wash buffer (10 mM Tris-HCl [pH 7.5], 1 mM EDTA, and 2 M NaCl) and biotinylated oligo-dT primer (IDT; 5′-/BiotinTEG/AAG CAG TGG TAT CAA CGC AGA GTA CTT TTT TTT TTT TTT TTT TTT TTT TTT TTT TVN-3′) at 100 µM for 30 minutes at room temperature on a rotator. The oligo-treated beads were washed four times in 1× binding & wash buffer (5 mM Tris-HCl [pH 7.5], 0.5 mM EDTA, and 1 M NaCl) and then suspended in 1× SuperScript II First Strand Buffer (Invitrogen) supplemented with SUPERaseIn RNase Inhibitor (Invitrogen) to a final concentration of 1 U/µL. The lysate was thawed on ice. 10 µL of prepared oligo-dT beads was added to each well containing 12 µl cell lysate using a Dragonfly Discovery liquid dispenser (SPT Labtech). The lysate plate was sealed and incubated on a ThermoMixer C (Eppendorf) with a heated lid at 21°C for 20 minutes, shaking at 1,000 rpm. Using a Fluent 480 liquid handling robot (Tecan) and a Magnum FLX magnetic separator (Alpaqua), the lysate supernatant was transferred to a new plate and the beads were washed twice in a custom wash buffer (50 mM Tris-HCl [pH 8.3], 75 mM KCl, 3 mM MgCl2, 10 mM DTT, and 0.5% Tween-20). The supernatant from the washes was added to the leftover cell lysate containing the genomic DNA, which was stored at −20°C overnight. The mRNA was not used for this study. The remaining cell lysate was thawed and subjected to a 0.6× vols Ampure XP clean-up with 80% ethanol. The bead-bound gDNA was isothermally amplified for 3 hours at 30°C then 10 minutes at 65°C using a miniaturized (1/5 vols) Repli-g Single-Cell assay (Qiagen). The amplified gDNA was cleaned up with 0.8× vols Ampure XP and 80% ethanol and then eluted in 10 mM Tris-HCl.

Sequencing libraries for this project were constructed by the Technical Genomics Team at the Earlham Institute, Norwich, UK, using the KAPA High-Throughput Library Prep Kit (Roche Part No: KK8234/07961901001). Where possible, 1 µg of genomic DNA was sheared to 450 bp using the Covaris ML230 Sonicator (Covaris), and the ends of the DNA were repaired; 3′ to 5′ exonuclease activity removed the 3′ overhangs, and the polymerase activity filled in the 5′ overhangs, creating blunt ends. A single “A” nucleotide was added to the 3′ ends of the blunt fragments to allow for the ligation of barcoded adapters (6 bp—Perkin Elmer NEXTFLEX DNA Barcodes 1–48 [NOVA-514101/2/3/4]) or (12 bp—Perkin Elmer NEXTFLEX-HT [NOVA-51474/5/6/7]) at a concentration of 6 µM prior to a 0.8× clean up using Beckman Coulter AMPure XP beads (A63882). The size of the libraries was estimated using an Agilent High Sensitivity DNA chip (5067-4626), and the concentrations were quantified by fluorescence with a High-Sensitivity Qubit assay (ThermoFisher Q32854). Libraries were sequenced on an Illumina NovaSeq 6000 (PL0398) or an Illumina NovaSeq X Plus (PL0403) to produce 150 bp paired-end reads.

### Genome assembly

Sequencing reads were trimmed using Trim Galore (version 0.6.6) (https://github.com/FelixKrueger/TrimGalore) with Cutadapt (version 3.4) (41). Genome assemblies were generated using SPAdes (version 3.15.5) (42) with single-cell mode enabled (--sc) and k-mer sizes 21, 33, 55, and 77. *Naegleria* and their bacterial cobiont genomes were separated based on a combination of taxonomic classification using CAT (version 5.3) (43), BlobTools (version 1.1.1) (44), Tiara (version 1.0.1) (45), metagenome binning with MetaBat2 (version 2.15) (46), and manual curation. Mitochondrial genomes and extrachromosomal rRNA plasmids were assembled and circularized separately using NOVOPlasty (version 4.3.1) (47). Mitochondrial genomes were annotated using MFannot (v1.35) (48) and visualized using OGDRAW (49).

### Genome annotation

Protein-coding genes were predicted as follows for the genomes of *Naegleria* sp. PL0398, *Naegleria* sp. PL0403, *Neovahlkampfia damariscottae* (22), and *Tetramitus jugosus* CCAP 1588/3C (23). *De novo* repeat libraries were generated using RepeatModeler (version 2.0.5) (50), and assemblies were soft-masked using RepeatMasker (version 4.1.7) (51). Initial gene sets were predicted using GeneMark-EP (52), with hints generated by ProtHint from a protein database that included the proteomes of *Acrasis kona* (24), *Naegleria clarki* (8), *Naegleria fowleri* (53), *Naegleria gruberi* (5), and *Naegleria lovaniensis* (7). These initial gene sets were filtered by selecting complete gene models based on full-length sequence alignments against the same protein database above using Diamond (version 2.0.15) in ultra-sensitive mode (54). These filtered gene sets were used as training genes to train an Augustus (version 3.5.0) model for each species (55). Final gene sets were generated using the species-specific Augustus model for each species with the hints generated by ProtHint. Completeness was assessed using BUSCO (v5.7.0) (56) in protein mode with the “eukaryota_odb10” data set. Protein domains were annotated using InterProScan (version 5.63-95.0) (57) with the Pfam database (release 35.0) (58). Signal peptides were predicted using SignalP (version 6.05) with the fast model (59).

### *Naegleria* 18S rRNA phylogenetics

18S rRNA genes were annotated using barrnap (version 0.9) (https://github.com/tseemann/barrnap) with “kingdom” set to “euk.” Multiple sequence alignment of 18S rRNA sequences from the *Naegleria* genus and an outgroup sequence from *Willaertia magna* was performed using MAFFT (version 7.271) with the G-INS-I algorithm (60). Maximum likelihood phylogenetic reconstruction was performed using IQ-TREE (version 2.3.6) (61). The best-fitting model (TPM3 +R2) was determined using ModelFinder (62), and support was assessed with 200 non-parametric bootstrap replicates.

### Heterolobosean phylogenomics and comparative genomics

Phylogenomic analysis was performed using our BUSCO_phylogenomics pipeline (https://github.com/jamiemcg/BUSCO_phylogenomics), which identified 152 BUSCO proteins from the eukaryota_odb10 that were complete and single-copy in at least nine out of 10 Heterolobosea species. Each BUSCO family was individually aligned using MUSCLE (version 5.1) (63), trimmed with trimal (version 1.5) (64) in “automated1” mode, and then concatenated together, resulting in a supermatrix alignment 76,279 amino acids in length. A Bayesian species phylogeny was constructed using PhyloBayes-MPI (version 1.8) (65) under the CAT-GTR model. Two independent Markov and Monte Carlo chains were run for approximately 8,000 generations. Convergence was assessed using bpcomp and tracecomp, with a burn-in of 20%. The phylogeny was rooted with *Neovahlkampfia damariscottae* as an outgroup. Phylogenies were visualized and edited using iTOL (66). Orthogroups were identified using OrthoFinder (version 2.5.5) (67) with Diamond in ultra-sensitive mode and the flag “-M msa” set to infer maximum likelihood trees from multiple sequence alignments. Singleton orthogroups (i.e., unassigned genes) were included in our analyses of orthogroups. UpSetR (68) was used to generate the UpSet plot visualizing orthogroup conservation. Orthogroups were mapped onto the species phylogeny, and gains and losses were inferred using the Dollo parsimony algorithm implemented in Count (version 10.04) (69).

### *Legionella* phylogenomics and comparative genomics

Genome assemblies for 166 species representatives from the family Legionellaceae were downloaded from the GTDB database (accessed October 2024) (70), along with the genome of *Coxiella burnetti,* which served as an outgroup. Completeness and contamination statistics were determined using CheckM2 (version 1.0.2) (71) with default parameters. Bacterial genomes were annotated using Prokka (version 1.14.6) (72). Protein domains were annotated using InterProScan (version 5.63-95.0) (53) with the Pfam database (release 35.0) (54). Protein structures were predicted using AlphaFold 3 (73) and aligned using Foldseek (74). The T4Sepp pipeline (75) was used to predict effectors, and subcellular localization was predicted using DeepLoc (v2.1) (76).

BUSCO proteins were identified using BUSCO (version 5.7.0) (56) in protein mode with the “legionellales_odb10” data set. A phylogenomic species tree was reconstructed using our BUSCO_phylogenomics pipeline (https://github.com/jamiemcg/BUSCO_phylogenomics) which identified 184 BUSCO proteins that were complete and single-copy in at least 94% of species (i.e., at least 159 out of 169 bacterial genomes) which were individually aligned using MUSCLE (version 5.1) (63), trimmed with trimal (version 1.5) (64) in “automated1” mode, and then concatenated together resulting in a supermatrix alignment 47,837 amino acids in length. A partitioned maximum-likelihood phylogenomic analysis was performed using IQ-TREE (version 2.3.6) (61). The partitioning scheme and best fitting models were determined using ModelFinder (62), which partitioned the 184 proteins into 34 partitions. Support was assessed with 1,000 SH-aLRT and 1,000 ultrafast bootstrap replicates (77). The resulting phylogeny was visualized and annotated using iTOL (66). Orthogroups were identified using OrthoFinder (version 2.5.5) (67) with Diamond in ultra-sensitive mode and the flag “-M msa” set to infer maximum likelihood trees from multiple sequence alignments.

We manually annotated Dot/Icm genes in each bacterial genome by blastp (78) searches of *L. pneumophila* Dot/Icm components against the predicted protein sets. Synteny plots were visualized using gggenomes (79). We also performed automated annotation of secretion systems across all bacterial genomes in our data set using MacSyFinder (v2.1.4) (32) with the TXSScan (v1.1.3) (80) models in “ordered_replicon” mode. We ignored MacSyFinder pT4SSi annotations given the divergent organization of the Dot/Icm secretion system relative to other T4SS. HipBST toxin-antitoxin systems were manually identified by blastp searches using *L. pneumophila* HipBST proteins as queries. The HipBST system was only considered present if each protein was encoded in a gene cluster.

## Data Availability

The raw sequencing reads and annotated *Naegleria* and *Legionella* genome sequences were deposited on the European Nucleotide Archive under BioProject PRJEB85689. Additional supporting data have been deposited on Zenodo (10.5281/zenodo.15721720).

## SUPPLEMENTAL MATERIAL

Figure S1 msphere.00352-25-s0001.pdf GC content and sequencing coverage of *Naegleria* sp. PL0398 and *Naegleria* sp. PL0403.

Figure S2 msphere.00352-25-s0002.pdf Circular maps of the mitochondrial genomes and the extrachromosomal rDNA plasmids of *Naegleria* sp. PL0398 and *Naegleria* sp. PL0403.

Figure S3 msphere.00352-25-s0003.pdf Maximum-likelihood phylogenetic reconstruction of *Naegleria* 18S rRNA sequences using IQ-TREE under the TPM3+R2 model.

Figure S4 msphere.00352-25-s0004.pdf Predicted structure of putative TAL-like effector from *Legionella quateirensis* color coded by AlphaFold pLDDT scores.

Table S1 msphere.00352-25-s0005.xlsx Presence or absence of BUSCO genes across Heterolobosean genomes.

Table S2 msphere.00352-25-s0006.xlsx *Naegleria fowleri* orthogroup gains and losses.

Table S3 msphere.00352-25-s0007.xlsx *Naegleria* and *Willaertia* antistasin-like domain proteins.

Table S4 msphere.00352-25-s0008.xlsx *Legionella* secretion systems and HipBST toxin-antitoxin system.

Table S5 msphere.00352-25-s0009.xlsx Eukaryotic InterPro domains found in *Legionella* genomes.

Table S6 msphere.00352-25-s0010.xlsx Putative TAL-like effectors found in *Legionella* genomes.

ASM does not own the copyrights to Supplemental Material that may be linked to, or accessed through, an article. The authors have granted ASM a non-exclusive, world-wide license to publish the Supplemental Material files. Please contact the corresponding author directly for reuse.
