# Supplementary material for: Comparative single-cell genomics of two uncultivated Naegleria species harboring Legionella cobionts
Source: mSphere. 2025 Aug 27;10(9):e00352-25. doi: 10.1128/msphere.00352-25 (PMC12482156; doi:10.1128/msphere.00352-25)
Supplement: Figure S1 — GC content and sequencing coverage of Naegleria sp. PL0398 and Naegleria sp. PL0403. [file msphere.00352-25-s0001.pdf]

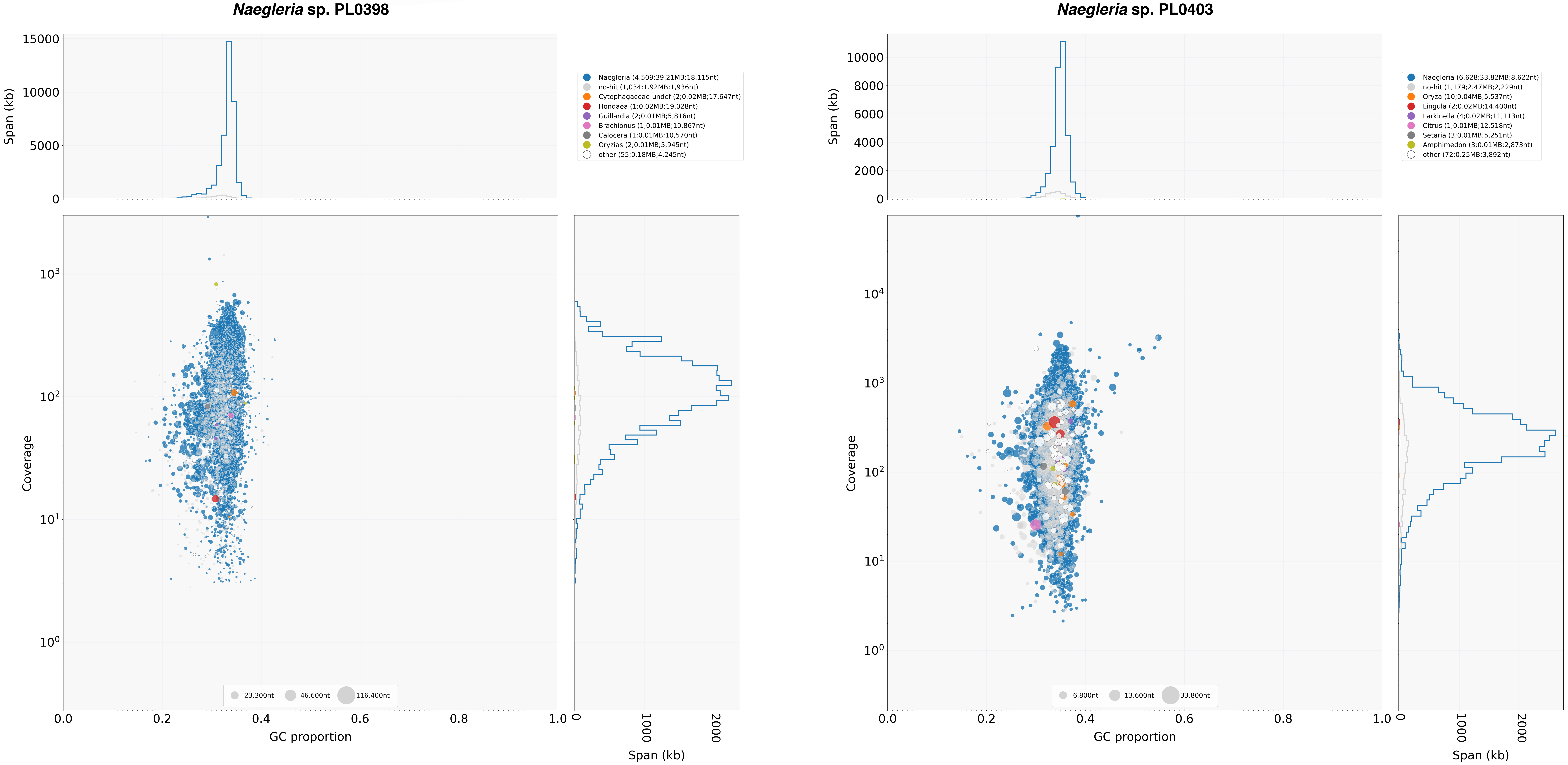

Figure S1. Blob plots showing the % GC content (horizontal axis) and sequencing coverage (vertical axis) of *Naegleria* sp. PL0398 and *Naegleria* sp. PL0403. Scaffolds are represented as dots with their size proportional to sequence length and coloured according to taxonomic classification. In both cases, approximately 99% of each assembly is classified as *Naegleria* or unclassified, with the remaining sequences only having spurious hits to diverse unrelated taxa.
