# Supplementary material for: Comparative single-cell genomics of two uncultivated Naegleria species harboring Legionella cobionts
Source: mSphere. 2025 Aug 27;10(9):e00352-25. doi: 10.1128/msphere.00352-25 (PMC12482156; doi:10.1128/msphere.00352-25)
Supplement: Figure S2 — Circular maps of the mitochondrial genomes and the extrachromosomal rDNA plasmids of Naegleria sp. PL0398 and Naegleria sp. PL0403. [file msphere.00352-25-s0002.pdf]

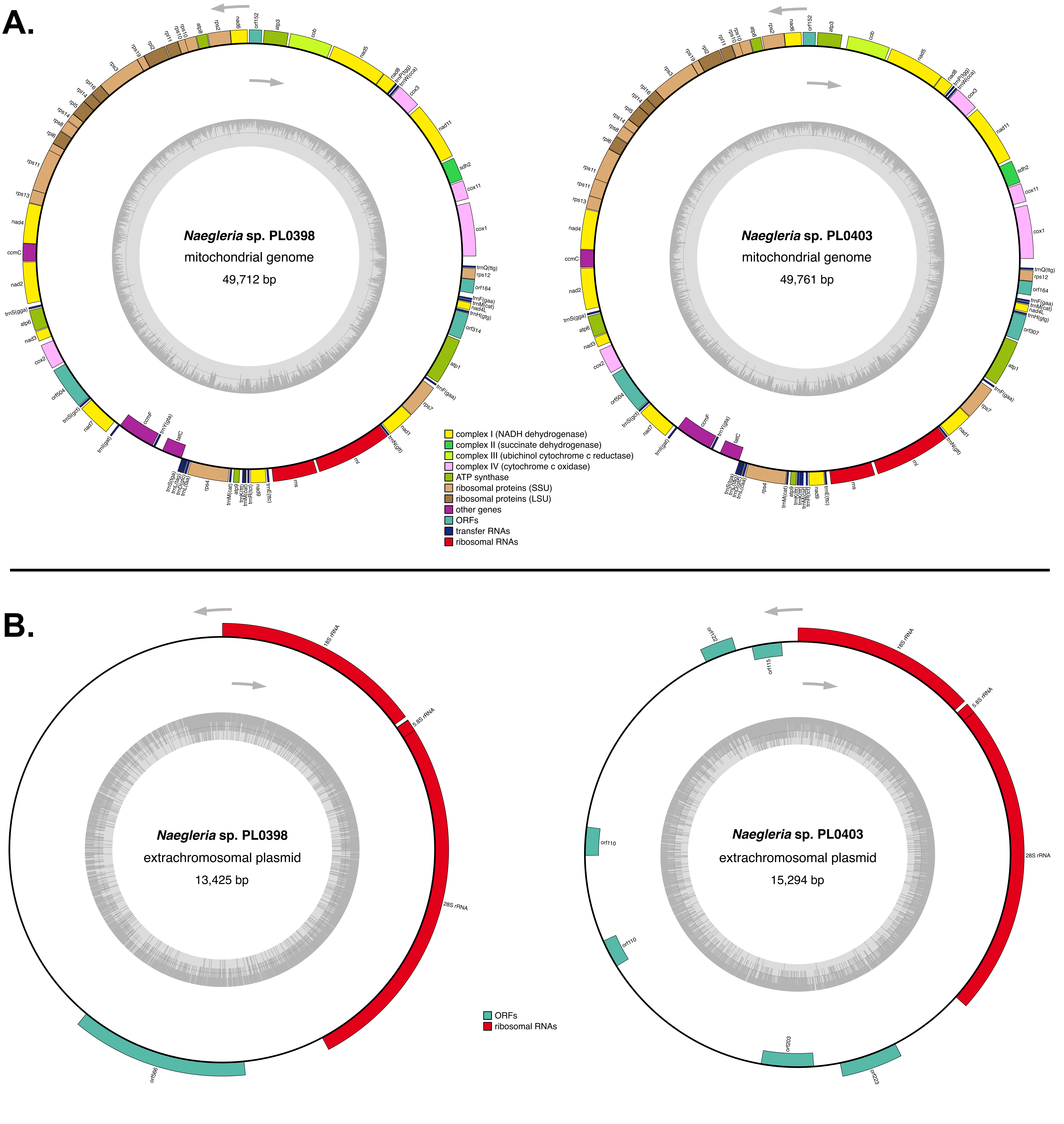

Figure S2. (A) Circular maps of the mitochondrial genomes of *Naegleria* sp. PL0398 and *Naegleria* sp. PL0403. Arrows indicate the direction of transcription. The inner ring shows GC content. **(B)** Circular maps of the extrachromosomal rDNA plasmids of *Naegleria* sp. PL0398 and *Naegleria* sp. PL0403. Arrows indicate the direction of transcription. The inner ring shows GC content.
