# Supplementary material for: Comparative single-cell genomics of two uncultivated Naegleria species harboring Legionella cobionts
Source: mSphere. 2025 Aug 27;10(9):e00352-25. doi: 10.1128/msphere.00352-25 (PMC12482156; doi:10.1128/msphere.00352-25)
Supplement: Figure S3 — Maximum-likelihood phylogenetic reconstruction of Naegleria 18S rRNA sequences using IQ-TREE under the TPM3+R2 model. [file msphere.00352-25-s0003.pdf]

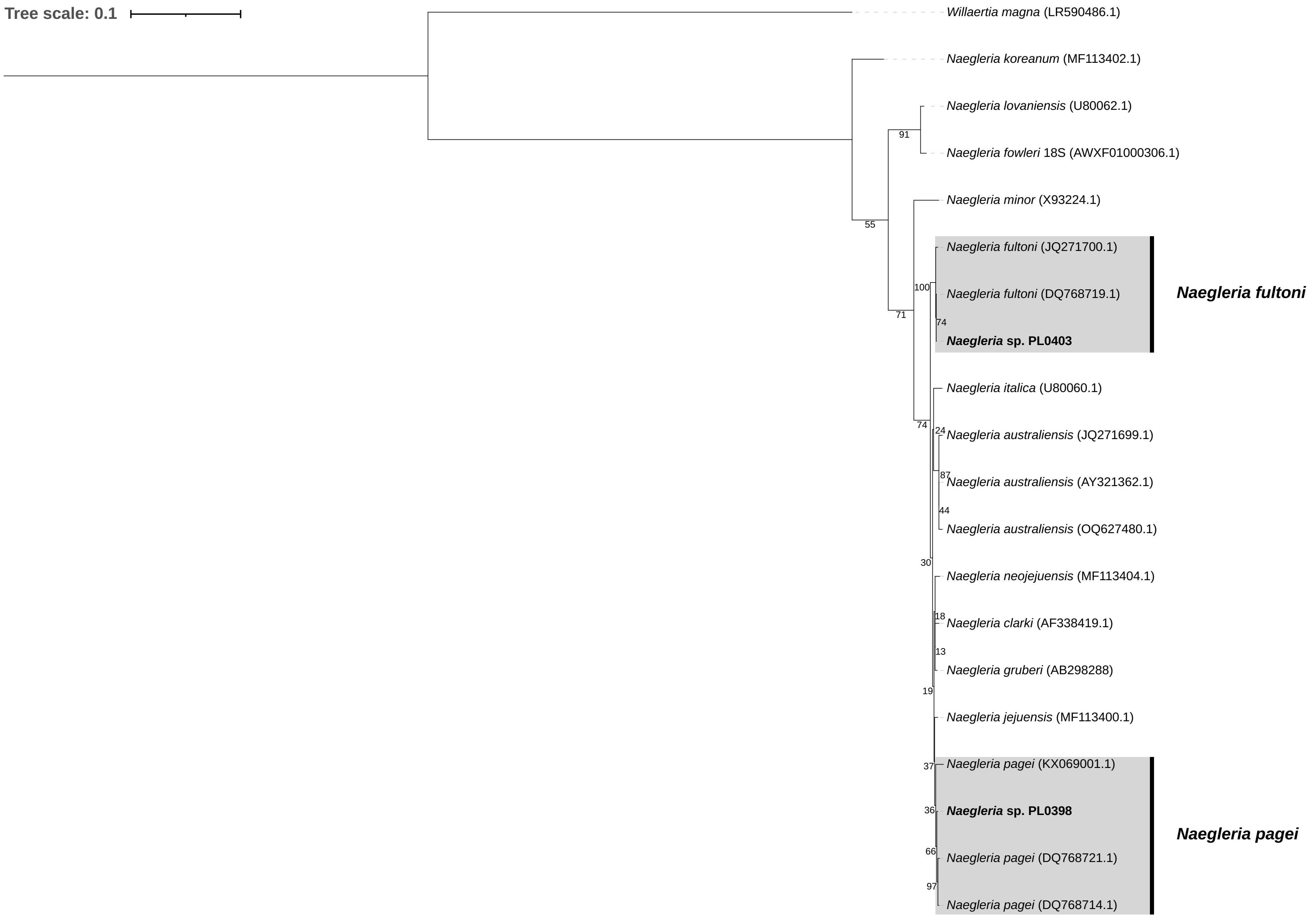

Figure S3. Maximum-likelihood phylogenetic reconstruction of *Naegleria* 18S rRNA sequences using IQ-TREE under the TPM3+R2 model. Numbers along branches indicate support from 200 non-parametric bootstrap replicates. *Willaertia magna* was included as an outgroup.
