# Supplementary material for: Comparative single-cell genomics of two uncultivated Naegleria species harboring Legionella cobionts
Source: mSphere. 2025 Aug 27;10(9):e00352-25. doi: 10.1128/msphere.00352-25 (PMC12482156; doi:10.1128/msphere.00352-25)
Supplement: Figure S4 — Predicted structure of putative TAL-like effector from Legionella quateirensis color coded by AlphaFold pLDDT scores. [file msphere.00352-25-s0004.pdf]

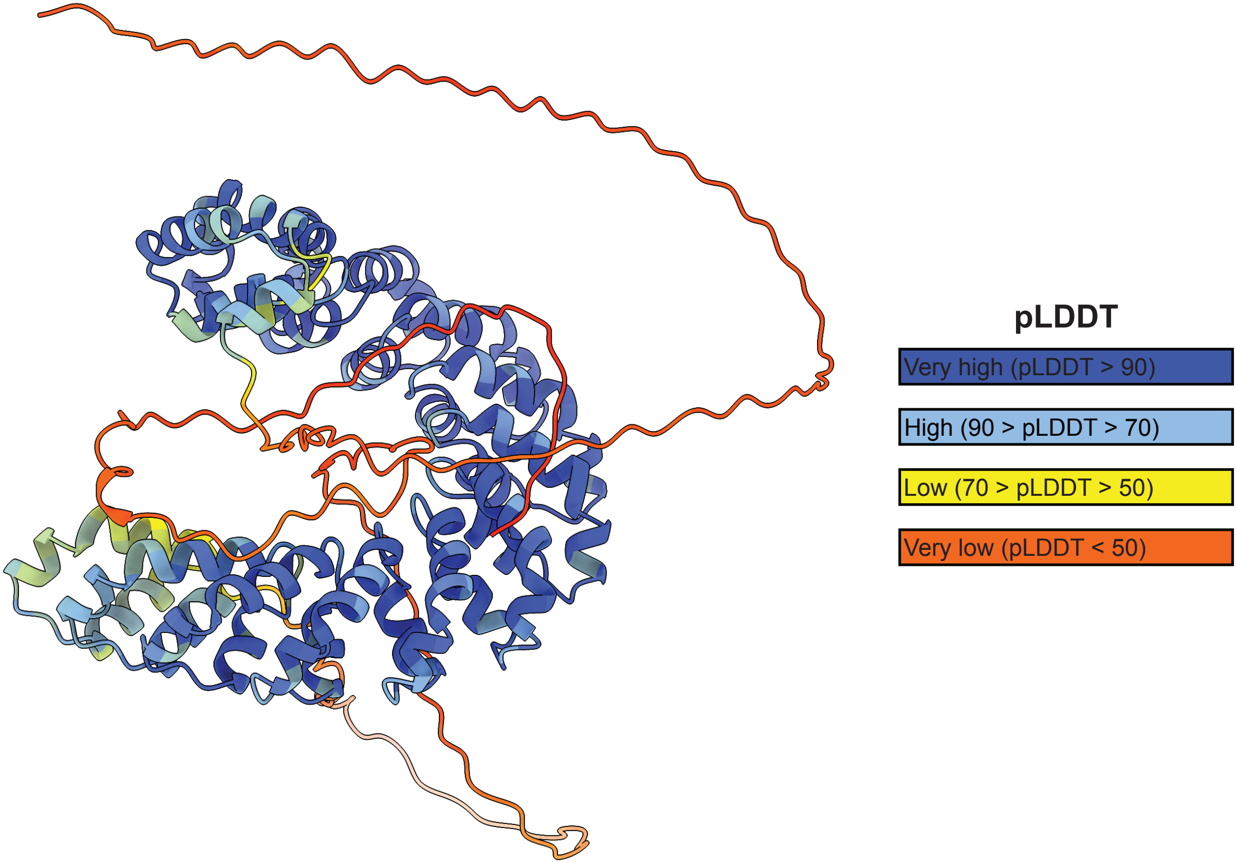

Figure S4. Predicted structure of putative TAL-like effector from Legionella quateirensis colour coded by AlphaFold pLDDT scores. The core of the protein structure is predicted with high to very high confidence based on pLDDT but the N and C-termini have low pLDDT scores.
